# 2418. Incidence and Characteristics of Midline-Associated Bloodstream Infections in an Urban Healthcare System

**DOI:** 10.1093/ofid/ofad500.2038

**Published:** 2023-11-27

**Authors:** Rebecca Choudhury, Jordan Ehni, Itay Rabinovitz, Nikita Ekhelikar, Waleed Javaid, Bernard Camins, Mateen Jangda

**Affiliations:** Mount Sinai Queens Hospital, Astoria, New York; Mount Sinai Beth Israel, Brooklyn, NY; Mount Sinai Morningside, West and Beth-Israel, New York, New York; Icahn School of Medicine at Mount Sinai, New York, New York; Mount Sinai Health System, New York, NY; Icahn School of Medicine at Mount Sinai, New York, New York; Georgia Institute of Technology, Marietta, Georgia

## Abstract

**Background:**

Midline catheters may carry a lower infection risk than central venous catheters, but available data is limited. Central line-associated bloodstream infection (CLABSI) incidence has increased during the COVID-19 pandemic, but is unclear what influence the pandemic has had on midline-associated bloodstream infections (MABSI). The objective of this study was to determine the incidence of MABSI during the COVID-19 pandemic and which pathogens were involved.

**Methods:**

A health system infection prevention database was used to identify adult patients admitted to 6 acute care hospitals in 2021 who had a midline during admission and a positive blood culture. Cases were reviewed by a hospital epidemiologist and 3 supervised trainees for demographic data, microbiology data, and MABSI attribution using National Healthcare Safety Network (NHSN) CLABSI definitions.

Midline-Associated Bloodstream Infection Identification

**Figure:**
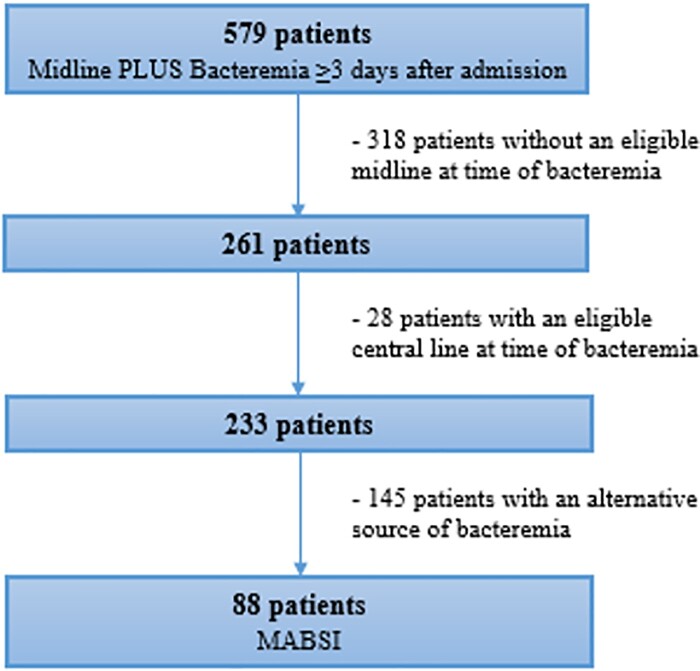

**Results:**

579 patients with possible MABSI were identified. 88 patients were determined to have a MABSI. 28 patients were excluded due to having a CLABSI-eligible central line at the time of the BSI. The remainder were excluded because the BSI did not fall within a MABSI eligibility period or because the BSI was secondary to another infection. MABSI and CLABSI rates for each hospital are shown in Table 1. MABSI occurred mostly in medicine patients (73, 82.95%). A slight majority of patients were male (48, 54.54%), and median age was 66 years (range 24-96). The most common organisms were *Candida* spp. (27), coagulase-negative *Staphylococcus* spp. (17), *Enterococcus* spp. (17), and *Staphylococcus aureus* (15). The 2021 yearly health system MABSI and CLABSI rates were similar (1.29 vs. 1.2), with variation between hospitals. Quarterly MABSI and CLABSI rates are shown in Figure 2. The health system MABSI rate appeared to increase during Winter 2021, while the CLABSI rate seemingly decreased.

MABSI and CLABSI Rates

**Figure:**
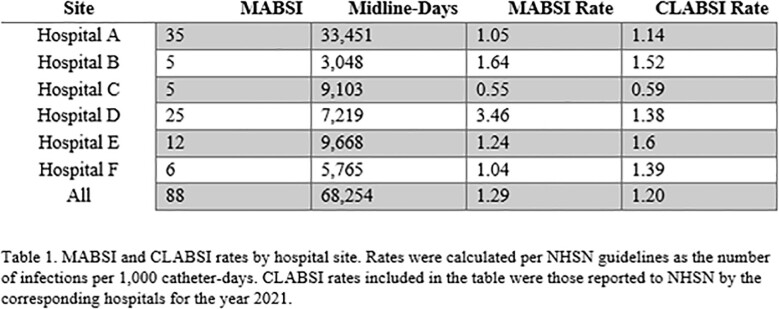

**Figure:**
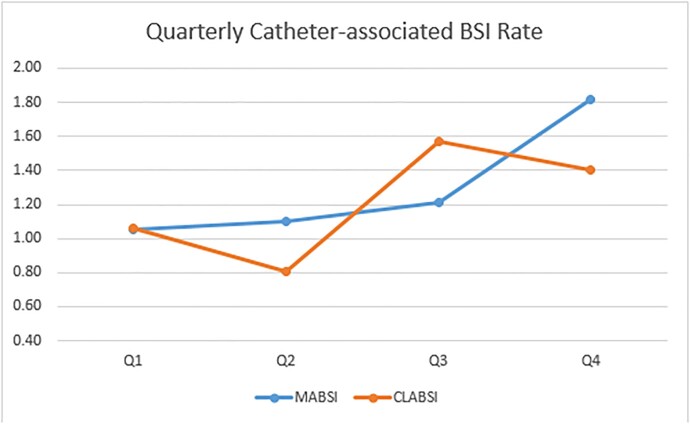

MABSI and CLABSI rates by quarter of 2021. The blue line represents the MABSI rate, while the orange line represents the CLABSI rate.

**Figure:**
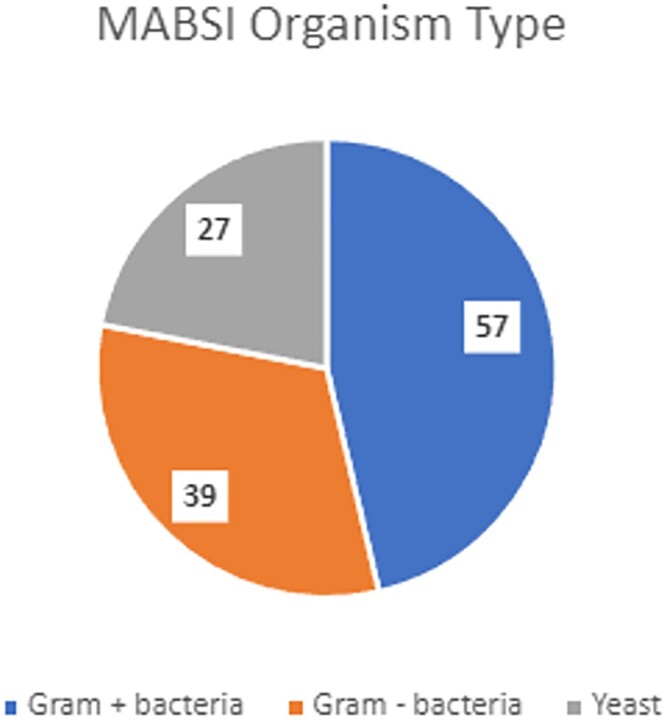

Organisms isolated from blood cultures in MABSI patients. Gram positive bacteria were the most common isolates (57/123, 46.34%). More than 88 organisms were detected as many MABSI involved ≥2 possible pathogens (27/88, 30.68%).

**Conclusion:**

In this study MABSI demographics were similar to those reported for CLABSI in other studies. While gram positive bacteria were common, as described in CLABSI, we also observed a high proportion of infections due to Candida spp. The health system MABSI rate was higher than the CLABSI rate, contrary to some studies. This was primarily driven by hospitals which were heavily impacted by the Omicron subvariant surge.

**Disclosures:**

**Bernard Camins, MD, MSc**, Puro Lighting, LLC: Advisor/Consultant

